# Peritonsillar and deep neck infections: a review of 330 cases^☆^

**DOI:** 10.1016/j.bjorl.2017.03.008

**Published:** 2017-04-09

**Authors:** Paula Martínez Pascual, Paloma Pinacho Martinez, Eviatar Friedlander, Carlos Martin Oviedo, Bartolome Scola Yurrita

**Affiliations:** Hospital General Universitario Gregorio Marañón, Madrid, Spain

**Keywords:** Deep neck infections, Mediastinitis, Peritonsillar infections, Complications, Antibiotics, Infecções cervicais profundas, Mediastinite, Infecções peritonsilares, Complicações, Antibióticos

## Abstract

**Introduction:**

Deep neck infections are defined as suppurative infectious processes of deep visceral spaces of the neck.

**Objective:**

The aim of this study is to review different factors that may influence peritonsillar and deep neck infections and may play a role as bad prognosis predictors.

**Methods:**

We present a retrospective study of 330 patients with deep neck infections and peritonsillar infections who were admitted between January 2005 and December 2015 in a tertiary referral hospital. Statistical analysis of comorbidities, diagnostic and therapeutic aspects was performed with Excel and SPSS.

**Results:**

There has been an increase in incidence of peritonsilar and deep neck infections. Systemic comorbidities such as diabetes or hepatopathy are bad prognosis factors. The most common pathogen was *S. viridans* (32.1% of positive cultures). 100% of the patients received antibiotics and corticosteroids, 74.24% needed surgical treatment. The most common complications were mediastinitis (1.2%) and airway obstruction (0.9%).

**Conclusion:**

Systemic comorbidities are bad prognosis predictors. Nowadays mortality has decreased thanks to multidisciplinary attention and improvements in diagnosis and treatment.

## Resumo

**Introdução:**

Infecções cervicais profundas são definidas como processos infecciosos supurativos dos espaços viscerais profundos do pescoço.

**Objetivo:**

O objetivo desse estudo é analisar diferentes fatores que podem influenciar as infecções peritonsilares e cervicais profundas que podem desempenhar um papel como preditores de mau prognóstico.

**Método:**

Apresentamos um estudo retrospectivo de 330 pacientes portadores de infecções cervicais profundas e de infecções peritonsilares admitidos entre janeiro de 2005 e dezembro de 2015 em um hospital terciário de referência. A análise estatística de comorbidades, aspectos diagnósticos e terapêuticos foi realizada utilizando-se os programas Excel e o SPSS.

**Resultados:**

Houve um aumento na incidência de infecções peritonsilares e infecções cervicais profundas. Comorbidades sistêmicas como diabetes ou doença hepática são fatores de mau prognóstico. O patógeno mais comum foi *S. viridans* (32,1% das culturas positivas). 100% dos pacientes receberam antibióticos e corticosteroides, e 74,24% necessitaram de tratamento cirúrgico. As complicações mais comuns foram mediastinite (1,2%) e obstrução das vias aéreas (0,9%).

**Conclusão:**

Comorbidades sistêmicas são preditores de mau prognóstico. Atualmente, a mortalidade diminuiu graças ao cuidado multidisciplinar e melhorias no diagnóstico e tratamento.

## Introduction

Deep Neck Infections (DNI) are defined as suppurative infectious processes of deep visceral spaces of the neck that usually originates as soft tissue fasciitis and may lead to an abscess.^1^ Direct extension of an upper aerodigestive infection through fascial planes is the most common cause. DNI are a frequent emergency in Otolaryngology which can be life-threatening as it may lead to airway obstruction, mediastinitis or jugular vein thrombosis.^2^ The aim of this study is to review different factors that may be the predisposing ones to an increase of infection risk and may have an important role in prognosis.

## Methods

We performed a retrospective study of patients diagnosed of cervical infection who were admitted in the emergency room of our hospital from January 2005 to December 2015. We excluded patients with superficial skin infections, limited intraoral infections (such us dental phlegmon or abscess) and cervical necrotizing fasciitis. Finally, 330 patients were enrolled in our study.

Although peritonsillar infections are not truly DNI, we decided to include them in our review because of its high incidence and sometimes coexistence with other deep neck space infection.

We used excel and SPSS to perform statistical analysis and Pearson X^2^ test were calculated to obtain *p*-values.

## Results

### Demography

**Figure 1:**
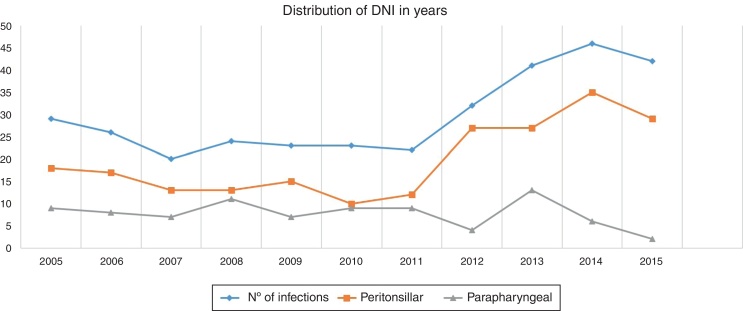
Distribution of DNI in years.

**Figure 2:**
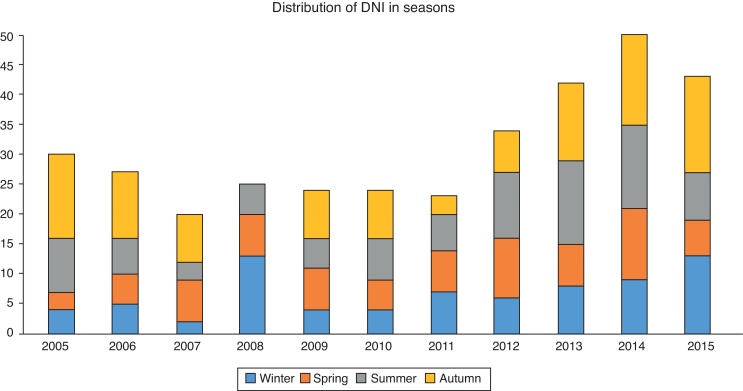
Distribution of DNI in seasons.

There were 176 men (53.3%) and 154 women (46.7%). Our population ages ranged from 6 months to 87 years, the mean age was 32.89 ± 18.198 years. 81.51% of them were adults (269 out of 330) and 19.49% were children (aged ≤ 16 years old). 50% were older than 31 years old. The mean number of patients with a neck infection admitted in our hospital per year during 11 years was 29.82 people. The distribution by years is shown separately in Fig. 1. Autumn was the period where more patients presented a DNI, 8.55 ± 4.82 cases (range 2–15 cases). This implies that between the end of September and the first half of December, 2.85 patients were admitted per month due to this pathology. The distribution in seasons is displayed in Fig. 2. The mean hospital stay was 4.54 days. 7.3% (24/330) of the population had allergy to some antibiotics, penicillin was the most common (18 patients) followed by aminoglycosides and quinolones. 62 patients (18.8%) had had previously a DNI, and 14 (4.2%) had had tonsillectomy done years before.

### Underlying systemic diseases

There were 28 patients with underlying systemic diseases (Table 1). Diabetes Mellitus (DM) was the most prevalent in our population.

**Table 1:** Underlying sistemic diseases.

Comorbidity	No. of patients (%) *n* = 330
Diabetes Mellitus	9 (2.7%)
Haematologic malignancies	5 (1.5%)
Hepatopathy	4 (1.2%)
Isquemic cardiomyopathy	3 (0.9%)
HIV	2 (0.6%)
Tuberculosis	2 (0.6%)
Chronic renal failure	2 (0.6%)
Neoplasm	1 (0.3%)
No comorbidity	302 (91.5%)

### Etiology

The etiology of the infection was identified in 296 patients (89.7%). The most common cause was pharyngotonsillar infections (277 cases, 83.9%), followed by odontogenic infections (11 cases, 3.3%). Rest of the causes exposed in Table 2.

**Table 2:** Etiology.

Origin of infection	No. of patients (%) *n* = 330
Pharyngotonsillar	277 (83.9%)
Dental	11 (3.3%)
Salivary glands	4 (1.2%)
Foreign bodies	2 (0.6%)
Previous cervical surgery	1 (0.3%)
Bacteriemia	1 (0.3%)
Non identified cause	34 (10.3%)

### Localization

The peritonsillar space was the most commonly affected. Distribution of localizations is shown in Table 3.

**Table 3:** Localization of infection.

Cervical space involved	No. of patients (%) *n* = 330
Peritonsillar	215 (65.2%)
Parapharyngeal	91 (27.6%)
Retropharyngeal	11 (3.3%)
Submandibular	3 (0.9%)
Base of tongue	3 (0.9%)
Ludwig angina	2 (0.6%)
Cervical anterior space	2 (0.6%)
Multiple space	3 (0.9%)

### Clinical presentation

The most common symptom reported by the patients was odynophagia in 98.2% of patients while the most common sign was the presence of trismus in 55.5%, followed by cervical lymphadenopathies in 53.6%.

### Previous treatment

244 patients (73.9%) had not received antibiotics prior to admission in our hospital. Those who have been treated had been taking penicillins in most of the cases (83.72%). The rest of the patients had received macrolides (16.28%), usually in a 3 day monodose treatment.

### Diagnosis

A Fine Needle Aspiration (FNA) was realized in 277 patients (73.9%), in 22.74% of the cases (63 patients) purulent material was obtained, classifying it as an abscess. In routinary blood test, abnormal blood cell count was found with an increase in neutrophils in 313 cases (94.8%).

When the physical examination and the FNA were not enough to reach a diagnose, an imaging technique was realized. Cervical CT with iodinated contrast was the gold standard, DNI was described as diffuse inflammation area (phlegmon) or a hypodense area with the presence of a “rind” (wall with a distinguishable inner and outer margin), an air/fluid level or scattered small gas bubbles (abscess). CT was needed in 194 cases (58.8%), and 48 of them required a second one due to a bad clinical evolution during hospital stay. Usually a second image test was performed after 48 h without any improvement with treatment. Cervical ecography was realized in 4 patients, they were one child under 1 year old and three adults with a severe renal failure in order not to expose them to iodinated contrast material. In two children with suspicion of retropharyngeal infection we preferred a cervical lateral radiography to avoid unnecessary radiation in infants. In these cases an increase of soft tissue in the retropharyngeal space was shown.

Bacterial cultures were just possible in 221 patients (66.96%) however a positive result was obtained in 61.99% of them (137 cases). The isolated pathogens and their incidence are shown in Table 4.

**Table 4:** Microorganisms distribution in patients with positive bacterial cultures.

Pathogen	No. of patients with positive bacterial culture (%) *n* = 137
*S. viridans*	44 (32.1%)
*S. pyogenes*	31 (22.6%)
Anaerobic bacteria	17 (12.4%)
Polymicrobe	11 (8.0%)
*Haemophilus* spp.	10 (7.3%)
*S. aureus*	7 (5.1%)
*Strepto* spp.	5 (3.6%)
*S. epidermidis*	3 (2.2%)
*A. odontolyticus*	3 (2.2%)
Gram negative bacteria (*Klebsiella* spp., *E. coli*, *Neisseriae* spp.)	4 (2.8%)
*Candida*	1 (0.7%)
*Mycobacterium tuberculosis*	1 (0.7%)

### Treatment

All of our patients received antibiotics and corticosteroids. In 304 cases (92.1%) we chose a β lactamic associated with an inhibitor of β lactamases. Those who were allergic to β lactamics (21 patients), were treated with an aminoglycoside (6 patients, 1.8%) or a quinolone (1 patient, 0.3%) in monotherapy or associated with an antibiotic against anaerobic microorganisms (e.g. clindamycin, metronidazole) (3 patients, 0.9%). There were three patients who required a drug change because of antibiotic resistance or torpid evolution; in those cases we preferred carbapenems. We had one DNI in our population caused by *Mycobacterium tuberculosis*, so it was treated with tuberculostatics drugs in the same way a respiratory infection is handled. Patients were treated with antibiotics during a mean time of 10.92 ± 3.73 days (range 4–35 d). Those who needed intensive care unit stay were the ones who required a longer antibiotic treatment.

245 of them (74.24%) needed surgical drainage, 196 (80%) needed a transoral approach while 36 (14.7%) required a cervicotomy. In 4 patients we opted for a combined approach (transoral and cervical incision), it was usually used in multispace infection when the affected area were not adjacent. When there was tonsillar necrosis or intratonsillar abscess (9 cases, 3.7%), we performed a tonsillectomy at the time of surgical drainage. 16 of 245 patients (6.53%) who had been operated on, needed a second surgery because of bad clinical evolution.

### Complications

13 of our patients (3.9%) had complications. Mediastinitis was the most frequent one (4 cases, 1.2%) followed by airway obstruction (3 cases, 0.9%), cellulitis, pneumonia (2 cases respectively, 0.6%), acute renal failure and sepsis (1 case respectively, 0.3%). Tracheostomy was performed in 6 patients, 3 of them due to acute airway compromise and the other 3 secondary to prolonged orotracheal intubation. We observed a vocal cord paralysis and a Horner syndrome in two patients after surgery. 5 of 13 required intensive care unit attentions, with a mean stay of 49 days (range 1–69 d). One patient died from septic shock.

The factors that were related with complications were analyzed. Male patients and those allergic to penicillins had a higher rate of complications and ICU stay. All factors are shown in Table 5.

**Table 5:** Comparison of distribution of factors associated with complications and ICU stay versus patients with no complications and no ICU stay.

	No. (%) of patients with complications		*p*-Value	No. (%) of patients who required ICU stay		*p*-Value
*Sex*	No	Yes		No	Yes	
Male	167 (94.9)	9 (5.1)	0.241	171 (97.2)	5 (2.8)	0.035
Female	150 (97.4)	4 (2.6)		154 (100)	0	

*Antibiotic allergy*						
None	296 (96.7)	10 (3.3)	0.140	303 (99)	3 (1)	0.038
Penicillin	15 (83.3)	3 (16.7)		16 (88.9)	2 (11.1)	
Other antibiotic	6 (100)	0		6 (100)	0	

*Comorbidity*						
No	293 (97)	9 (3)	<0.0001	300 (99.3)	2 (0.7)	<0.0001
DM	8 (88.9)	1 (11.1)		8 (88.9)	1 (11.1)	
Hepatopathy	1 (25)	3 (75)		2 (50)	2 (50)	
Other comorbidity	15 (100)	0		15 (100)	0	
Microorganisms	No	Yes		No	Yes	
*S. viridans*	42 (95.5)	2 (4.5)	<0.0001	43 (97.7)	1 (2.3)	<0.0001
Polymicrobe	8 (72.7)	3 (27.3)		9 (81.8)	2 (18.2)	
*S. pyogenes*	31 (100)	0		31 (100)	0	
Others	240 (98.4)	4 (1.6)		243 (99.6)	1 (0.4)	
Etiology						
Unknown	31 (91.2)	3 (8.8)	0.199	33 (97.1)	1 (2.9)	0.523
Pharyngotonsillar	269 (97.1)	8 (2.9)		274 (98.9)	3 (1.1)	
Dental	10 (90.9)	1 (9.1)		10 (90.9)	1 (9.1)	
Salivary glands	3 (75)	1 (25)		4 (100)	0	
Foreign bodies	2 (100)	0		2 (100)	0	
Previous cervical surgery	1 (100)	0		1 (100)	0	
Bacteriemia	1 (100)	0		1 (100)	0	

## Discussion

In our review pharyngotonsillar infections were the most common cause of peritonsillar and DNI. This result is consistent with some studies in the literature,^1^, ^2^, ^3^, ^4^ although for the majority, odontogenic infections are the main cause, especially in studies carried out in Asia and Eastern Europe.^5^, ^6^, ^7^ This may be related to different oral hygiene conditions between different countries. Although peritonsillar infections are not strictly DNI we chose to consider them in our review, as well as other studies did,^2^, ^6^, ^8^ because in many cases it was the start of a proper DNI or because it had severe complications as a DNI can have. If we quantified just strict DNI we had a population of 91 parapharyngeal and 11 retropharyngeal infections in 10 years. In patients with DNI is more common to find cases who had not had a tonsillectomy, this may be explained as tonsils have an increased bacterial load living within crypts.^7^

We would like to enhance that we found an increase in DNI incidence in the second period studied (2011–2015); this could be due to an aging population or the fear to over prescribe antibiotics and develop resistant microorganisms. In fact, 3 out of 4 people had not taken any medication prior to the emergency consult.

In this study we found that systemic comorbidities like diabetes mellitus^3^, ^4^, ^9^, ^10^ or hepatopathy and allergy to penicillins are common in cases of DNI who suffer complications or require ICU stay. DM results in a defect of polymorphonuclear neutrophil function, cellular immunity and complement activation. Consequently, hyperglycemia and high glycosylated hemoglobin are predictors of worse prognosis,^10^ due to it, our diabetic patients (including those unknown who present with high blood glucose concentration while hospitalization) were studied by the Endocrinology department. The prevalence of penicillin allergy in our review (5.5%) was lower than the global population one (10%–12%),^11^ however it was much higher in patients who required ICU stay (2 out of 5) or who suffered complications (3 out of 13).

*S. viridans* was the most common pathogen in our population, as well as in other studies.^3^, ^6^, ^12^ We did not find *Klebsiella pneumoniae* in our environment, which differs from studies in Asia.^4^, ^8^, ^10^ They usually find a high prevalence of this microorganism, specially in diabetic patients.^13^ We had two ways of obtaining material for culture, either a FNA in the consult or a sample obtained during surgical drainage. Sometimes none of them could be performed. Some patients had a severe trismus which hindered the FNA (it was realized in 227/330 cases). On the other hand, 245 patients (74.24%) received surgical drainage, which is the best moment to take a sample of the infected material, but it was not always possible, as in some cases the material obtained from the infected area was not enough or was not in suitable conditions (purulent material mixed with blood or saliva). Besides, even when the sample was enough cultures were not always positive. This may be explained by antibiotics taken prior to sample extraction or an incorrect sample management (e.g. anaerobes in an aerobic environment).

According to the treatment, we confirm what most studies have already said. Every patient received antibiotics and corticosteroids.^1^, ^2^, ^3^, ^4^, ^5^, ^6^, ^7^, ^8^, ^9^, ^10^, ^11^, ^14^ Surgical drainage still is the option when medical treatment is not enough, when there is already a well formed hypodense area with margins well defined or an air/fluid level or signs of complications such as mediastinitis or involvement of multiple regions.^15^

Complications may appear as a consequence of extension of the infection through neck spaces. Mediastinitis and airway obstruction were the most common ones as previous studies have shown before.^1^, ^2^, ^3^ In cases of mediastinitis, thoracic surgeons performed the drainage in the same surgical time as our team did a cervicotomy. Traqueostomy was needed in a lower percentage than other studies,^3^ around 1% like some Indian review.^6^ The use of corticosteroids decreases tissue edema and the probability of pus gush into the airway while endotracheal intubation, making the procedure safer and more successful.^16^, ^17^

Even there has been an increase in DNI incidence, mortality remains low as it have been previously shown in other studies.^17^, ^18^, ^19^

## Conclusions

DNI are still common and can develop serious complications. Immunocompromised patients with systemic comorbidities are susceptible of worse prognosis. In spite of the increase in DNI, mortality has decreased thanks to multidisciplinary attention and improvements in imaging techniques or antibiotics and surgery, which have enabled an earlier diagnosis and treatment.

## Conflicts of interest

The authors declare no conflicts of interest.
